# Trends in atherosclerotic heart disease-related mortality among U.S. adults aged 35 and older: A 22-year analysis

**DOI:** 10.1016/j.ijcrp.2025.200374

**Published:** 2025-02-10

**Authors:** Muzamil Akhtar, Danish Ali Ashraf, Muhammad Salman Nadeem, Ayesha Maryam, Hasan Ahmed, Mehmood Akhtar, Sarah MaCKenzie Picker, Raheel Ahmed

**Affiliations:** a Gujranwala Medical College, Gujranwala, Pakistan; b Foundation University Medical College, Islamabad, Pakistan; c Nishtar Medical College, Nishtar Medical University, Multan, Pakistan; d Imperial College London, London, UK; e Bolan Medical College, Quetta, Pakistan; f Sunderland Royal Hospital, Sunderland, UK; g National Heart and Lung Institute, Imperial College London, UK

**Keywords:** Atherosclerotic heart disease, Cardiovascular diseases, Mortality trends, CDC WONDER

## Abstract

**Background:**

Atherosclerotic heart disease (ASHD) remains a leading cause of mortality worldwide, especially among older adults. Understanding the long-term mortality trends in ASHD can guide public health strategies and address demographic disparities.

**Methods:**

Mortality data for individuals aged 35 years and older were extracted from the CDC WONDER database. Age-adjusted mortality rates (AAMR) per 100,000 persons were calculated and stratified by year, gender, race, urbanization, and place of death. The trends were assessed using the annual percent change (APC) and average annual percent change (AAPC) with 95 % confidence intervals (CI) calculated through Joinpoint regression analysis.

**Results:**

From 1999 to 2020, 7,638,608 ASHD-related deaths were recorded. The overall AAMR declined from 291.08 in 1999 to 170.07 in 2020, with an AAPC of −2.70 % (95 % CI: 2.96 to −2.54). However, an abrupt rise was observed from 2018 to 2020 (APC: 4.55; 95 % CI: 0.77 to 6.75). Males reported higher AAMR than females (Males: 271.9 vs. Females: 151.9). Non-Hispanic (NH) White individuals had the highest AAMR (209.38), followed by NH Black (202.47), NH American Indian (176.12), Hispanic (158.1), and NH Asian (113.7) populations. Nonmetropolitan areas reported the highest AAMR (214.77), while medium metropolitan areas reported the lowest (195.41). The majority of deaths occurred in medical facilities (42.81 %), followed by decedent's homes (25.67 %), and nursing homes (24.79 %).

**Conclusion:**

Despite a long-term decline in ASHD-related mortality, the recent increase from 2018 to 2020 requires further study. Gender and racial disparities persist, highlighting the need for targeted public health efforts to reduce these inequities.

## Graphical abstract

**Figure:**
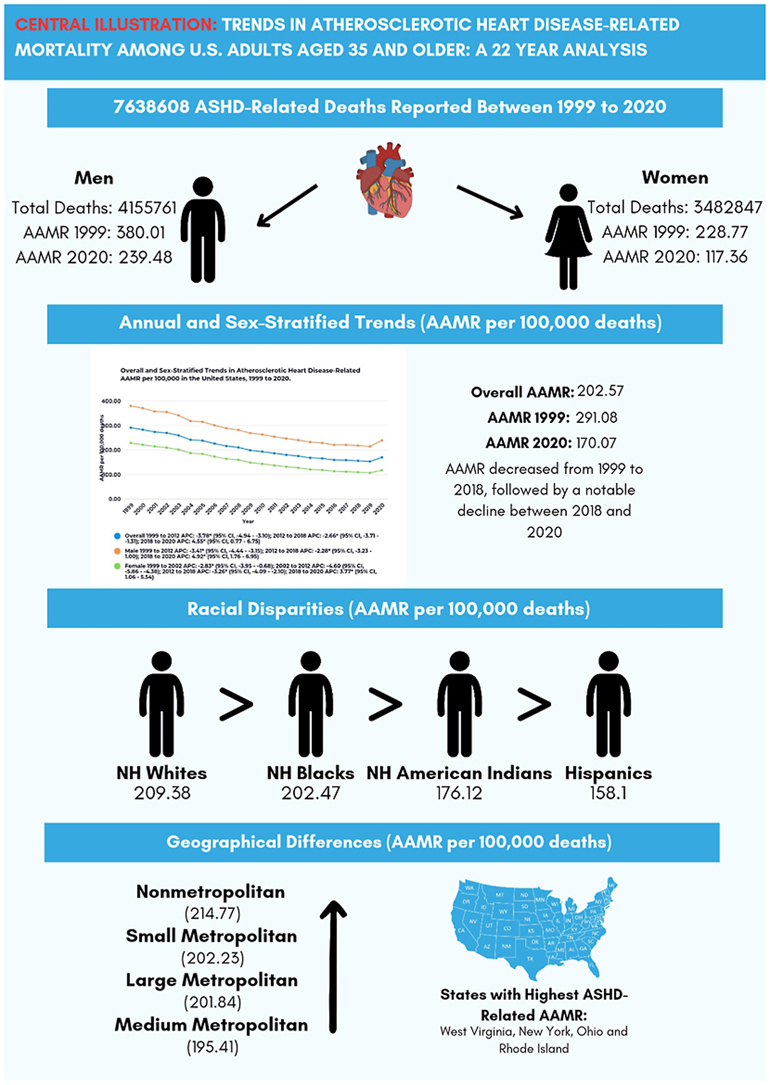

## 1 Introduction

Atherosclerosis, a leading global vascular disease, underlies various serious health conditions, including coronary artery disease (CAD), peripheral artery disease (PAD), and cerebrovascular/carotid artery disease [1,2]. This progressive disorder is characterized by endothelial damage, low-grade inflammation, lipid accumulation, and plaque formation within the intima of the blood vessel wall [3]. Atherosclerotic disorders are the main contributors to the burden and trends of cardiovascular disease (CVD) [1]. According to estimates from the Global Burden of Disease study, the global prevalence of CAD reached 315 million in 2002 [4].

Over the past 25 years, sociodemographic changes have led to significant declines in CVD in regions with very high Socio-Demographic Index (SDI). In contrast, in many other regions, the decline has been slower or even minimal [5]. According to a study by Fowkes et al., global populations are experiencing a significant epidemiological transition, with the burden of atherosclerotic cardiovascular diseases (ASCVD) rapidly shifting from high-income countries to low- and middle-income countries [6].

As the leading cause of mortality and morbidity worldwide, ASCVD imposes a significant medical and economic burden on society [7]. Understanding the contributing factors is crucial for developing effective prevention and intervention strategies. The aim of this study is to analyze the demographic and regional trends in the atherosclerotic heart disease (ASHD)-related mortality among US adults aged 35 and older from 1999 to 2020. Ultimately, this analysis serves as a crucial tool for detecting high-risk populations and guiding the implementation of interventions to reduce deaths related to ASHD.

## 2 Methodology

### 2.1 Study design and population

This study analyzed death certificate data from the CDC WONDER database spanning the years 1999–2020 [8]. The primary focus was to examine mortality trends associated with Atherosclerotic Heart Disease (ICD-10-I25.1) among individuals aged 35 years and older. Since the dataset is publicly accessible and de-identified, the study did not require institutional review board approval. The study followed the STROBE (Strengthening the Reporting of Observational Studies in Epidemiology) guidelines [9].

### 2.2 Data collection

Data were extracted and categorized based on demographic variables such as gender, race/ethnicity, urbanization, states, and place of death. The locations of death included homes, nursing or long-term care facilities, medical facilities, hospices, and other unclassified locations. Racial and ethnic groups were classified as Non-Hispanic (NH) American Indian, NH Asian, NH Black or African American, NH White, and Hispanic or Latino. The geographic regions were defined using the National Center for Health Statistics Urban-Rural Classification Scheme [10].

### 2.3 Statistical analysis

The study assessed mortality trends related to atherosclerotic heart disease from 1999 to 2020, considering variables such as gender, race, urbanization, and states. Crude and age-adjusted mortality rates (AAMR) per 100,000 individuals were calculated, using the 2000 US population as the standard for AAMR. Annual percent change (APC) values with 95 % CI in AAMR were then calculated using the Joint point Regression Program (Join point V 4.9.0.0, National Cancer Institute). Not only did this allow us to quantify national trends, but also adjusted log-linear regression models wherever temporal variations occurred. This method allows for the identification of significant changes in AAMR over time. Using 2-tailed t-testing, APCs were deemed to be increasing or decreasing if the slope characterizing the change in mortality was significantly different from zero. P < 0.05 was taken to be statistically significant.

## 3 Results

From 1999 to 2020, ASHD accounted for 7,638,608 deaths in the U.S., with 4,155,761 in males and 3,482,847 in females (Supplementary Table 1). The majority of deaths occurred in medical facilities (42.81 %), followed by decedent's homes (25.67 %) and nursing homes (24.79 %).

### 3.1 Annual trends

**Fig. 1:**
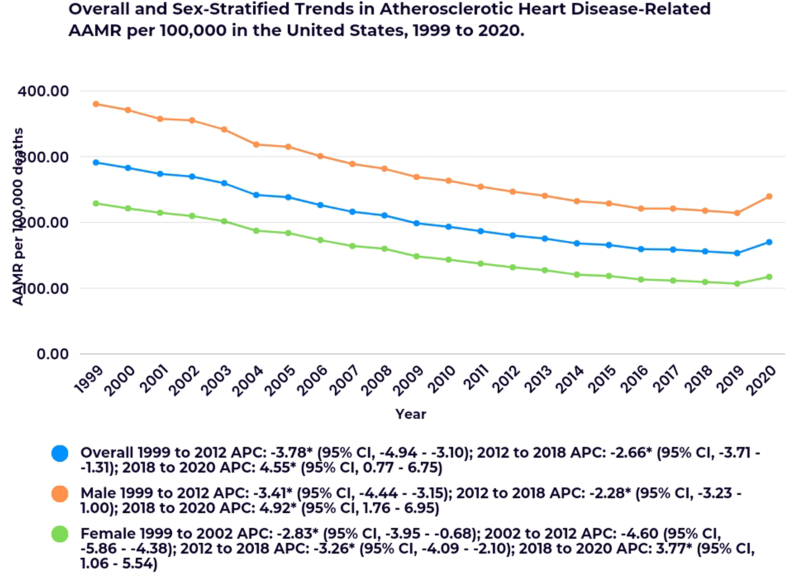
Overall and sex-stratified trends in atherosclerotic heart disease-related AAMR per 100,000 in the United States from 1999 to 2020. ∗Indicates that the Annual Percent Change (APC) is significantly different from zero at the alpha = 0.05 level.

The AAMR for ASHD declined from 291.08 per 100,000 in 1999 to 170.07 per 100,000 in 2020, with an AAPC of −2.70 (95 % CI, −2.96 to −2.54). The AAMR decreased significantly from 1999 to 2012 (APC: 3.78; 95 % CI, −4.94 to −3.10) and from 2012 to 2018 (APC: 2.66; 95 % CI, −3.71 to −1.31). However, there was an abrupt rise in the AAMR from 2018 to 2020, with an APC of 4.55 (95 % CI, 0.77 to 6.75) (Fig. 1; Supplementary Table 2).

### 3.2 AHD-related mortality stratified by demographics

#### 3.2.1 Gender-stratified trends

Males exhibited a higher overall AAMR as compared to females (Males AAMR: 271.9 vs. Females AAMR: 151.9) (Supplementary Table 1).

In females, AAMR declined during three distinct periods: 1999–2002 (APC: 2.83; 95 % CI, −3.95 to −0.68), 2002–2012 (APC: 4.60; 95 % CI, −5.86 to −4.38), and 2012–2018 (APC: 3.26; 95 % CI, −4.09 to −2.10). However, an increase in AAMR was observed from 2018 to 2020 (APC: 3.77; 95 % CI, 1.06 to 5.54).

In males, AAMR showed a decrease throughout the study period with an abrupt increase from 2018 to 2020 (APC: 4.92; 95 % CI, 1.76 to 6.95) (Fig. 1; Supplementary Table 3).

#### 3.2.2 Race-stratified trends

When stratified by race or ethnicity, NH White individuals exhibited the highest AAMR (209.38), followed by NH Black (202.47), NH American Indian (176.12), Hispanic (158.1), and NH Asian (113.7) populations (Supplementary Table 1).

Among NH White individuals, the AAMR decreased significantly from 1999 to 2012 (APC: 3.61∗; 95 % CI, −4.53 to −3.35) and continued to decrease from 2012 to 2018 (APC: 2.39∗; 95 % CI, −3.45 to −1.13). However, a subsequent increase was observed from 2018 to 2020 (APC: 3.89∗; 95 % CI, 0.51 to 5.92).

Similarly, in the NH Black population, the AAMR decreased throughout the study period, with a notable decline from 2011 to 2018 (APC: 2.99∗; 95 % CI, −3.86 to −1.41). This trend was reversed by a sharp increase from 2018 to 2020 (APC: 7.55∗; 95 % CI, 3.74 to 9.85).

NH American Indian individuals experienced a consistent decline in AAMR over the entire study period (APC: 1.90∗; 95 % CI, −2.30 to −1.46).

Among the Hispanic population, the AAMR also decreased significantly from 1999 to 2018 (APC: 4.32∗; 95 % CI, −4.63 to −4.04), followed by a notable increase from 2018 to 2020 (APC: 11.85∗; 95 % CI, 6.04 to 15.13).

**Fig. 2:**
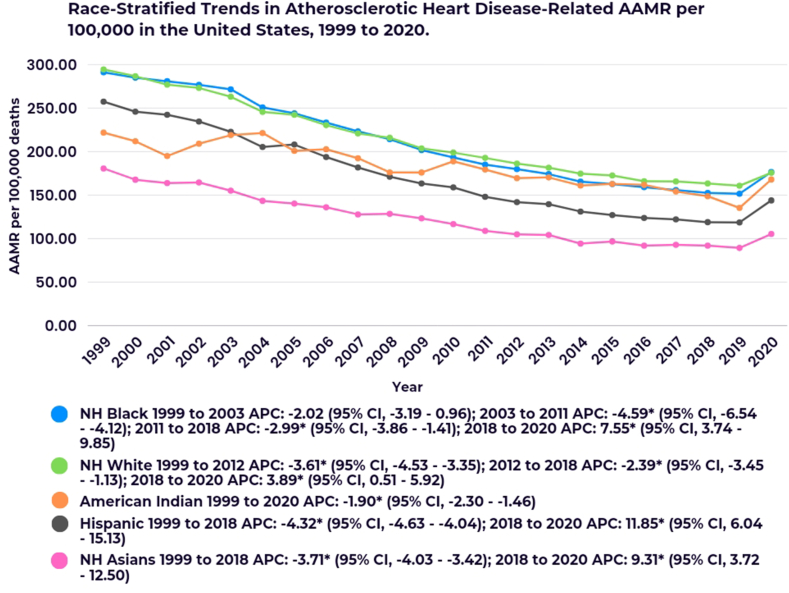
Race-stratified trends in atherosclerotic heart disease-related AAMR per 100,000 in the United States from 1999 to 2020. ∗Indicates that the Annual Percent Change (APC) is significantly different from zero at the alpha = 0.05 level.

In NH Asian populations, a decline in AAMR was observed from 1999 to 2018 (APC: 3.71∗; 95 % CI, −4.03 to −3.42). This was succeeded by a prominent increase from 2018 to 2020 (APC: 9.31∗; 95 % CI, 3.72 to 12.50) (Fig. 2; Supplementary Table 4).

#### 3.2.3 Urbanization-based differences

Nonmetropolitan areas reported the highest AAMR (214.77), followed by small metropolitan (202.23), large metropolitan (201.84), and medium metropolitan areas (195.41) (Supplementary Table 1).

In nonmetropolitan areas, a decrease in AAMR was observed from 1999 to 2018, followed by a prominent increase from 2018 to 2020 (APC: 4.89∗; 95 % CI, 0.87–7.10).

In small metropolitan areas, AAMR decreased consistently from 1999 to 2017 (APC: 2.89∗; 95 % CI, −3.20 to −2.79) before increasing from 2018 to 2020 (APC: 3.51∗; 95 % CI, 0.89–8.01).

In large metropolitan areas, AAMR decreased significantly from 1999 to 2018, with the most notable decline occurring between 1999 and 2014 (APC: 4.38∗; 95 % CI, −5.28 to −3.43). However, this trend reversed with an increase from 2018 to 2020 (APC: 4.20∗; 95 % CI, 0.43–6.72).

**Fig. 3:**
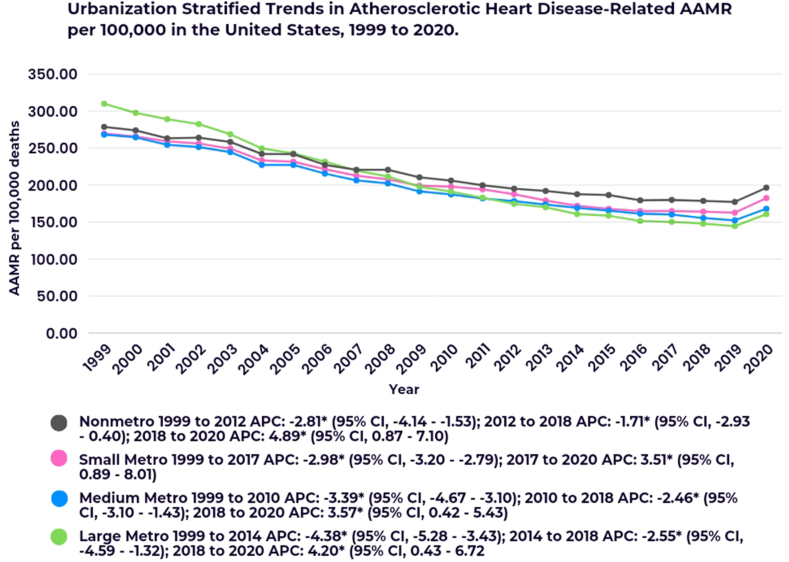
Urbanization-stratified trends in atherosclerotic heart disease-related AAMR per 100,000 in the United States from 1999 to 2020. ∗Indicates that the Annual Percent Change (APC) is significantly different from zero at the alpha = 0.05 level.

In medium metropolitan areas, AAMR decreased during two distinct periods: from 1999 to 2010 (APC: 3.39∗; 95 % CI, −4.67 to −3.10) and from 2010 to 2018 (APC: 2.46∗; 95 % CI, −3.10 to −1.43). This was followed by an increase from 2018 to 2020 (APC: 3.57∗; 95 % CI, 0.42–5.43) (Fig. 3; Supplementary Table 5).

#### 3.2.4 State-based differences

**Fig. 4:**
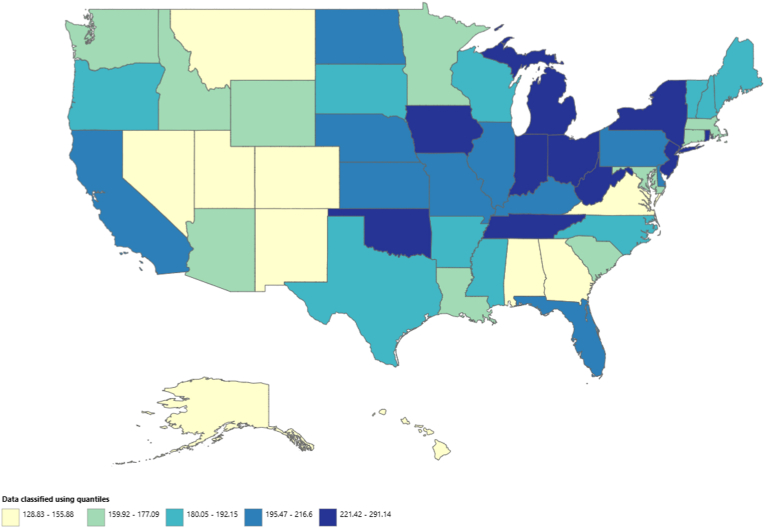
State-wise map highlighting the states with highest mortality in the United States from 1999 to 2020.

The AAMR varied significantly among states, ranging from 128.83 (95 % CI, 125.45–132.2) in Alaska to 291.14 (95 % CI, 289.07–293.21) in West Virginia. States in the top 90th percentile for AAMR included West Virginia, New York, Ohio, and Rhode Island, with rates more than double those in the bottom 10th percentile, which comprised Alaska, Hawaii, the District of Columbia, and Nevada. The highest percentage of total deaths occurred in California (11.17 %), followed by New York (9.49 %) and Florida (7.93 %) (Fig. 4; Supplementary Table 6).

## 4 Discussion

This study analyzed 22 years (1999–2020) of ASHD mortality trends in U.S. adults aged 35 and older using CDC WONDER data. Our findings have important public health implications. We observed a steady decline in AAMR from 1999 to 2018, followed by a sharp increase from 2018 to 2020. The decline may be attributed to greater awareness of modifiable risk factors (smoking, obesity, and physical inactivity) and advances in acute treatment and secondary prevention [11]. Public health campaigns have helped reduce smoking, which is a major risk factor for AHD [12]. However, it must be noted that despite the decrease in AAMR for AHD, there has been an increase in the crude mortality rate for AHD. This disparity can be explained by the booming global population. The sudden increase in AAMR from 2018 to 2020 can be due to an increase in the aging population. Moreover, contemporary lifestyle factors such as strained relationships, psychological stress and chronic sleep deprivation can also underpin this increase [13,14]. Studies have also highlighted the role of COVID-19 as a risk factor for cardiovascular diseases such as AHD [15,16], which corroborates the sharp increase in mortality from 2018 to 2020. The high inflammatory load and increased levels of circulating IL-6 as a result of cytokine storm during COIVD-19 infection can underly the precipitation of cardiovascular events [16,17]. This high mortality rate due to AHD presses for a mass public health initiative to decrease the risk factors associated with AHD and hence reduce the enormous burden on the healthcare system.

Upon stratifying the data on the basis of gender, the data elucidated a higher AAMR in males as compared to females. The male sex is a well-established risk factor for cardiovascular diseases, including AHD [18]. Furthermore, our analysis also revealed the racial disparity in the AAMR for AHD, with Whites having the highest mortality rate, followed by Blacks. It is well-understood that Blacks are at a higher risk for cardiovascular disorders than Whites, but we believe that a higher AAMR among Whites could be due to oversampling [19,20]. Nevertheless, the racial disparity underscores the need for tailored treatment regimens and further clinical research to better understand underlying mechanisms.

In addition, our analysis also revealed a regional disparity in AAMR for AHD. In the context of urbanization, nonmetropolitan areas showed the highest mortality rate, followed sequentially by small and large metropolitans. It has been shown by many studies that unhealthy lifestyles such as smoking, obesity, and lack of activity are more prevalent in rural areas, putting them at a greater risk for cardiovascular complications like AHD [[21], [22], [23]]. Furthermore, nonmetropolitan areas are not equipped with state-of-the-art medical facilities, especially those found in the large metropolitan areas, which further contributes to the increased mortality rate [24]. The people in nonmetropolitan areas are also disadvantaged in terms of education, socioeconomic conditions, and transportation as compared to their urban counterparts [25]. Public health policies focusing on reduction of smoking and obesity, and better healthcare access can help bridge the gap in mortality rates between nonmetropolitan and metropolitan areas. Along with this, there is a need for region-based studies to better understand such disparities and reduce the inequality in mortality rates between urban and rural areas.

Appropriate public health policies and measures can help mitigate the rural-urban divide in terms of access to healthcare facilities and mortality outcomes. The introduction of telemedicine facilities has been shown to improve healthcare access, quality and costs [26]. However, these improvements have been balanced out by closure of hospitals in the rural areas. Studies have also shown that despite intensive public health campaigns, the rural-urban divide has not shown significant improvements [27]. This calls for an extensive analysis of the underlying reasons for the widening gap so that the root of the problem can be addressed and subsequently resolved.

A significant state-based difference in mortality was also observed in our analysis with West Virginia and New York showing the highest values of AAMR. Mountaintop removal mining for coal has increased in the Central Appalachian region ever since the 1970s which has increased the particulate matter content of the air in the region [28]. New York has also experienced an increase in air pollution and particulate matter content. This increase in air pollution and particulate matter content has been associated with increased cardiovascular complications in many studies therefore contributing to the increased mortality in these regions. The dire implications of air pollution highlighted by our findings necessitates strict policies for reducing global emissions and reducing particulate matter content in the environment.

AHD ranks as the most prevalent cardiovascular disease and is a threat to the sustainable development in the 21st century [29]. In the US, the economic burden for AHD is significant, accounting for approximately 1%–1.5 % of the gross domestic product, with individual episodes costing up to $5000 [30]. Hence, there is a stringent need for public health measures to counter the rapidly increasing trend in mortality observed from 2018 onwards. This shall not only reduce the medical and economic burden but will also contribute to effective sustainable development.

### 4.1 Limitation

Our analysis has several limitations to consider. The main limitation is innate to the CDC WONDER database, which uses death certificates. These crucial statistics are subject to human error, underreporting of diseases, inaccurate evaluation of the cause of death, loss of data, or compilation errors. Additionally, people with AHD are also highly likely to have other comorbidities which can contribute to the death. Misclassification can also occur in the death certificates and reporting bias can result in underreporting of AHD as a cause of death. Secondly, the use of ICD-10 alone, without accompanying clinical information such as the drugs prescribed and other risk factors, restricts our ability to delve deeper into clinical associations. Thirdly, this population-based study lacks vital individual data comorbidities, prior treatments or interventions and duration of disease; all of which are important confounders of mortality. The failure to eliminate these confounding variables adds bias to the study. Nonetheless, our study still substantiates the demographic and temporal relationship between AHD over the past 22 years in the US and holds the potential to guide future public health policies.

## 5 Conclusion

To summarize, the AAMR for ASHD in the United States declined from 1999 to 2018 but showed a sharp increase from 2018 to 2020. Males, NH Whites, and individuals in nonmetropolitan areas experienced the highest mortality rates. These findings highlight the need for targeted public health interventions to address cardiovascular disparities and improve access to preventive care. Further research is warranted to explore the underlying drivers of these trends, including socioeconomic factors, healthcare access, and the long-term impact of COVID-19 on ASHD mortality.

## CRediT authorship contribution statement

**Muzamil Akhtar:** Writing – original draft, Formal analysis, Data curation, Conceptualization. **Danish Ali Ashraf:** Writing – original draft, Data curation, Conceptualization. **Muhammad Salman Nadeem:** Writing – original draft. **Ayesha Maryam:** Writing – original draft. **Hasan Ahmed:** Writing – original draft. **Mehmood Akhtar:** Writing – original draft. **Sarah MaCKenzie Picker:** Writing – review & editing. **Raheel Ahmed:** Writing – review & editing, Supervision.

## Consent to publish

Not Applicable.

## Ethics statement

No ethics approval was required for this study.

## Consent to participate

Not applicable.

## Funding

This research did not receive any specific grant from funding agencies in the public, commercial, or not-for-profit sectors.

## List of abbreviations:

**AAMR**: Age-Adjusted Mortality Rate
**APC**: Annual Percentage Change
**AAPC**: Average Annual Percentage Change
**ASHD**: Atherosclerotic Heart Disease
**CDC WONDER**: Centers for Disease Control and Prevention's Wide‐Ranging Online Data for Epidemiologic Research
