# Supplementary material for: Trends in atherosclerotic heart disease-related mortality among U.S. adults aged 35 and older: A 22-year analysis
Source: Int J Cardiol Cardiovasc Risk Prev. 2025 Feb 10;24:200374. doi: 10.1016/j.ijcrp.2025.200374 (PMC11872109; doi:10.1016/j.ijcrp.2025.200374)
Supplement: Multimedia component 1 [file mmc1.docx]

**Supplementary File:**

| **Table 1:** Frequency and age adjusted mortality rates per 100,000 deaths in individuals aged 35+ in the United States, 1999 to 2020 | | | |
| --- | --- | --- | --- |
|  | **Deaths** | **Population** | **Overall AAMR per 100,000 deaths (95% CI)** |
| Entire Cohort | 7638608 | 3553765020 | 202.57 (202.43 - 202.71) |
| ***Sex*** |  |  |  |
| Male | 4155761 | 1690398628 | 271.95 (271.68 - 272.21) |
| Female | 3482847 | 1863366392 | 151.94 (151.78 - 152.1) |
| ***Race*** |  |  |  |
| NH Asian or Pacific Islander | 152406 | 175362707 | 113.7 (113.12 - 114.28) |
| NH American Indians | 33565 | 25115894 | 176.12 (174.14 - 178.09) |
| Black or African American | 666366 | 394424863 | 202.47 (201.97 - 202.96) |
| NH White | 6359485 | 2550027482 | 209.38 (209.21 - 209.54) |
| Hispanic | 406497 | 408834074 | 158.1 (157.6 - 158.6) |
| ***2013 Urbanization*** |  |  |  |
| Large Metropolitan | 3892576 | 1932637216 | 201.84 (201.64 - 202.04) |
| Medium Metropolitan | 1565897 | 733379771 | 195.41 (195.1 - 195.72) |
| Small Metropolitan | 750357 | 326142460 | 202.23 (201.77 - 202.69) |
| Non-metropolitan | 1429778 | 561600310 | 214.77 (214.42 - 215.12) |

**Supplementary Table 1:** Frequency and age adjusted mortality rates per 100,000 deaths in adults aged 35+ in the United States, 1999 to 2020

| **Table 2:** Annual age adjusted mortality rates per 100,000 deaths in individuals aged 35+ in the United States, 1999 to 2020 | |
| --- | --- |
| **Year** | **Age Adjusted Mortality Rate per 100,000 deaths (95% CI)** |
| 1999 | 291.08 (290.19 - 291.98) |
| 2000 | 282.85 (281.97 - 283.73) |
| 2001 | 273.75 (272.89 - 274.6) |
| 2002 | 269.73 (268.88 - 270.57) |
| 2003 | 259.56 (258.74 - 260.38) |
| 2004 | 241.67 (240.89 - 242.46) |
| 2005 | 238.2 (237.43 - 238.98) |
| 2006 | 226.25 (225.51 - 227) |
| 2007 | 216.16 (215.43 - 216.88) |
| 2008 | 210.75 (210.05 - 211.46) |
| 2009 | 198.7 (198.02 - 199.38) |
| 2010 | 193.5 (192.84 - 194.17) |
| 2011 | 186.62 (185.97 - 187.26) |
| 2012 | 180.18 (179.56 - 180.81) |
| 2013 | 175.4 (174.79 - 176.01) |
| 2014 | 168.08 (167.48 - 168.67) |
| 2015 | 165.57 (164.99 - 166.15) |
| 2016 | 159.41 (158.85 - 159.98) |
| 2017 | 158.65 (158.09 - 159.21) |
| 2018 | 155.99 (155.45 - 156.54) |
| 2019 | 153.29 (152.76 - 153.83) |
| 2020 | 170.07 (169.52 - 170.63) |

**Supplementary Table 2:** Annual age adjusted mortality rates per 100,000 deaths in adults aged 35+ in the United States, 1999 to 2020

| **Table 3:** Age adjusted mortality rates per 100,000 deaths stratified by sex in individuals aged 35+ in the United States, 1999 to 2020 | | |
| --- | --- | --- |
|  | **Age Adjusted Mortality Rate per 100,000 deaths (95% CI)** | |
| **Year** | **Female** | **Male** |
| 1999 | 228.77 (227.76 - 229.77) | 380.01 (378.32 - 381.69) |
| 2000 | 221.31 (220.33 - 222.3) | 370.76 (369.11 - 372.41) |
| 2001 | 214.83 (213.87 - 215.79) | 357.39 (355.79 - 359) |
| 2002 | 209.85 (208.91 - 210.8) | 355.04 (353.46 - 356.63) |
| 2003 | 201.81 (200.89 - 202.74) | 341.22 (339.68 - 342.75) |
| 2004 | 187.48 (186.6 - 188.37) | 318.43 (316.96 - 319.9) |
| 2005 | 183.94 (183.07 - 184.81) | 315.02 (313.58 - 316.46) |
| 2006 | 173.11 (172.28 - 173.95) | 300.84 (299.46 - 302.23) |
| 2007 | 164.15 (163.34 - 164.95) | 288.85 (287.51 - 290.19) |
| 2008 | 159.99 (159.2 - 160.78) | 281.54 (280.23 - 282.84) |
| 2009 | 148.33 (147.57 - 149.09) | 269 (267.74 - 270.26) |
| 2010 | 143.42 (142.68 - 144.16) | 263.33 (262.09 - 264.56) |
| 2011 | 137.5 (136.79 - 138.22) | 254.19 (253 - 255.38) |
| 2012 | 131.81 (131.11 - 132.5) | 246.7 (245.54 - 247.86) |
| 2013 | 127.51 (126.83 - 128.19) | 240.62 (239.49 - 241.74) |
| 2014 | 120.62 (119.97 - 121.27) | 232.24 (231.15 - 233.33) |
| 2015 | 118.67 (118.03 - 119.32) | 228.75 (227.68 - 229.82) |
| 2016 | 113.29 (112.67 - 113.91) | 221.13 (220.09 - 222.16) |
| 2017 | 111.8 (111.19 - 112.42) | 221.11 (220.09 - 222.13) |
| 2018 | 109.42 (108.82 - 110.02) | 217.85 (216.85 - 218.85) |
| 2019 | 107.01 (106.42 - 107.6) | 214.32 (213.34 - 215.3) |
| 2020 | 117.36 (116.75 - 117.97) | 239.48 (238.45 - 240.5) |

**Supplementary Table 3:** Age adjusted mortality rates per 100,000 deaths stratified by sex in adults aged 35+ in the United States, 1999 to 2020

| **Table 4:** Age adjusted mortality rates per 100,000 deaths stratified by race in individuals aged 35+ in the United States, 1999 to 2020 | | | | | |
| --- | --- | --- | --- | --- | --- |
|  | **Age Adjusted Mortality Rate per 100,000 deaths (95% CI)** | | | | |
| **Year** | **NH Asian or Pacific Islander** | **NH Black or African American** | **NH White** | **Hispanic or Latino** | **NH American Indians or Alaska Native** |
| 1999 | 180.65 (175.5 - 185.8) | 291.13 (287.97 - 294.29) | 294.38 (293.4 - 295.35) | 257.34 (253.18 - 261.49) | 221.85 (208.64 - 235.06) |
| 2000 | 167.72 (162.95 - 172.5) | 285 (281.9 - 288.11) | 286.52 (285.56 - 287.48) | 245.82 (241.86 - 249.78) | 211.88 (199.59 - 224.17) |
| 2001 | 163.89 (159.37 - 168.41) | 280.83 (277.77 - 283.9) | 277.01 (276.07 - 277.95) | 242.27 (238.46 - 246.08) | 194.87 (183.26 - 206.49) |
| 2002 | 164.58 (160.2 - 168.97) | 276.66 (273.63 - 279.68) | 273.08 (272.15 - 274.01) | 234.49 (230.82 - 238.15) | 209.06 (197.06 - 221.06) |
| 2003 | 155.11 (150.98 - 159.24) | 271.58 (268.6 - 274.55) | 263.02 (262.11 - 263.92) | 222.69 (219.21 - 226.17) | 218.88 (206.72 - 231.05) |
| 2004 | 143.44 (139.6 - 147.28) | 250.7 (247.86 - 253.54) | 245.62 (244.75 - 246.49) | 205.21 (201.95 - 208.46) | 221.38 (209.21 - 233.56) |
| 2005 | 140.27 (136.62 - 143.92) | 243.96 (241.2 - 246.72) | 242.32 (241.47 - 243.18) | 208.06 (204.88 - 211.24) | 200.75 (189.44 - 212.06) |
| 2006 | 136.07 (132.58 - 139.57) | 233.3 (230.63 - 235.98) | 230.41 (229.58 - 231.24) | 193.82 (190.83 - 196.8) | 202.75 (191.53 - 213.97) |
| 2007 | 127.88 (124.59 - 131.16) | 223.28 (220.7 - 225.87) | 220.77 (219.96 - 221.57) | 181.7 (178.89 - 184.51) | 192.41 (181.65 - 203.16) |
| 2008 | 128.54 (125.35 - 131.73) | 214.31 (211.81 - 216.81) | 215.93 (215.14 - 216.72) | 171.18 (168.53 - 173.82) | 176.15 (166.15 - 186.14) |
| 2009 | 123.28 (120.24 - 126.32) | 201.85 (199.45 - 204.24) | 203.76 (203 - 204.53) | 163.43 (160.91 - 165.94) | 175.97 (166.11 - 185.83) |
| 2010 | 116.69 (113.8 - 119.58) | 193.29 (190.97 - 195.61) | 198.83 (198.08 - 199.58) | 158.88 (156.45 - 161.3) | 188.79 (178.69 - 198.89) |
| 2011 | 108.88 (106.21 - 111.54) | 185.13 (182.9 - 187.36) | 192.71 (191.97 - 193.44) | 147.98 (145.73 - 150.22) | 179.43 (169.87 - 188.99) |
| 2012 | 104.87 (102.34 - 107.39) | 179.95 (177.79 - 182.11) | 186.27 (185.56 - 186.99) | 141.93 (139.8 - 144.06) | 169.5 (160.56 - 178.45) |
| 2013 | 104.26 (101.84 - 106.68) | 174.11 (172.02 - 176.2) | 181.61 (180.91 - 182.31) | 139.56 (137.51 - 141.62) | 170.32 (161.57 - 179.06) |
| 2014 | 94.34 (92.12 - 96.56) | 165.55 (163.56 - 167.55) | 174.75 (174.07 - 175.43) | 131.17 (129.24 - 133.09) | 160.96 (152.77 - 169.15) |
| 2015 | 96.78 (94.61 - 98.95) | 162.62 (160.67 - 164.57) | 172.45 (171.78 - 173.13) | 126.98 (125.14 - 128.82) | 162.94 (154.88 - 171) |
| 2016 | 91.86 (89.81 - 93.92) | 159.04 (157.15 - 160.93) | 165.97 (165.31 - 166.63) | 123.85 (122.08 - 125.62) | 161.74 (153.92 - 169.57) |
| 2017 | 92.94 (90.94 - 94.94) | 155.72 (153.88 - 157.57) | 165.8 (165.15 - 166.45) | 122.14 (120.43 - 123.85) | 154.26 (146.8 - 161.71) |
| 2018 | 92.02 (90.08 - 93.96) | 152.61 (150.82 - 154.41) | 163.45 (162.82 - 164.09) | 118.93 (117.28 - 120.57) | 148.73 (141.64 - 155.81) |
| 2019 | 89.25 (87.39 - 91.11) | 151.57 (149.81 - 153.33) | 160.8 (160.17 - 161.43) | 118.67 (117.06 - 120.27) | 135.28 (128.65 - 141.9) |
| 2020 | 105.31 (103.35 - 107.28) | 176.62 (174.75 - 178.49) | 175.61 (174.95 - 176.26) | 143.91 (142.18 - 145.64) | 167.95 (160.76 - 175.14) |

**Supplementary Table 4:** Age adjusted mortality rates per 100,000 deaths stratified by race in adults aged 35+ in the United States, 1999 to 2020

| **Table 5:** Age adjusted mortality rates per 100,000 deaths stratified by urbanization in individuals aged 35+ in the United States, 1999 to 2020 | | | | |
| --- | --- | --- | --- | --- |
|  | **Age Adjusted Mortality Rate per 100,000 deaths (95% CI)** | | | |
| **Year** | **Large**  **Metropolitan** | **Medium**  **Metropolitan** | **Small**  **Metropolitan** | **Non-Metropolitan** |
| 1999 | 309.99 (308.69 - 311.3) | 268.31 (266.42 - 270.2) | 269.54 (266.78 - 272.3) | 278.78 (276.77 - 280.78) |
| 2000 | 297.71 (296.44 - 298.99) | 264.58 (262.72 - 266.45) | 265.91 (263.19 - 268.63) | 273.95 (271.97 - 275.94) |
| 2001 | 289.05 (287.81 - 290.3) | 254.44 (252.63 - 256.25) | 259.25 (256.58 - 261.91) | 263.4 (261.46 - 265.33) |
| 2002 | 282.56 (281.34 - 283.78) | 251.54 (249.76 - 253.33) | 256.21 (253.59 - 258.84) | 264.09 (262.16 - 266.02) |
| 2003 | 268.75 (267.57 - 269.93) | 244.69 (242.95 - 246.44) | 249.43 (246.86 - 252) | 258.42 (256.52 - 260.32) |
| 2004 | 249.64 (248.52 - 250.77) | 227.3 (225.63 - 228.96) | 233.37 (230.9 - 235.84) | 242.14 (240.31 - 243.98) |
| 2005 | 243.08 (241.98 - 244.18) | 227.25 (225.6 - 228.89) | 231.71 (229.28 - 234.14) | 241.92 (240.1 - 243.74) |
| 2006 | 231.64 (230.57 - 232.7) | 215.61 (214.03 - 217.2) | 221.63 (219.28 - 223.99) | 227.41 (225.66 - 229.17) |
| 2007 | 219.72 (218.7 - 220.75) | 206.39 (204.86 - 207.92) | 212.8 (210.52 - 215.08) | 220.7 (218.98 - 222.42) |
| 2008 | 211.72 (210.73 - 212.72) | 202.31 (200.81 - 203.82) | 207.77 (205.54 - 210) | 220.65 (218.94 - 222.36) |
| 2009 | 197.85 (196.9 - 198.8) | 191.46 (190.02 - 192.91) | 199.16 (197 - 201.32) | 210.43 (208.77 - 212.09) |
| 2010 | 191.19 (190.26 - 192.12) | 187.37 (185.95 - 188.79) | 197.86 (195.72 - 200) | 206.01 (204.37 - 207.64) |
| 2011 | 182.92 (182.02 - 183.82) | 182 (180.62 - 183.38) | 194.34 (192.24 - 196.44) | 199.83 (198.24 - 201.43) |
| 2012 | 174.85 (173.99 - 175.72) | 178.48 (177.13 - 179.83) | 187.6 (185.56 - 189.64) | 195.01 (193.44 - 196.57) |
| 2013 | 170.12 (169.28 - 170.96) | 173.82 (172.51 - 175.14) | 179.3 (177.33 - 181.27) | 192.08 (190.54 - 193.62) |
| 2014 | 160.72 (159.91 - 161.53) | 169.16 (167.87 - 170.45) | 171.96 (170.05 - 173.87) | 187.78 (186.26 - 189.3) |
| 2015 | 158.71 (157.91 - 159.5) | 165.42 (164.16 - 166.68) | 167.92 (166.05 - 169.79) | 186.64 (185.14 - 188.15) |
| 2016 | 151.46 (150.69 - 152.22) | 161.33 (160.1 - 162.56) | 164.76 (162.92 - 166.6) | 179.57 (178.11 - 181.04) |
| 2017 | 150.36 (149.6 - 151.11) | 160.19 (158.98 - 161.41) | 164.87 (163.05 - 166.69) | 179.88 (178.43 - 181.34) |
| 2018 | 147.85 (147.11 - 148.59) | 155.41 (154.23 - 156.58) | 164.19 (162.4 - 165.99) | 178.53 (177.09 - 179.97) |
| 2019 | 144.59 (143.87 - 145.31) | 152.37 (151.22 - 153.52) | 162.79 (161.02 - 164.56) | 177.31 (175.89 - 178.74) |
| 2020 | 160.76 (160 - 161.51) | 168.01 (166.81 - 169.21) | 182.37 (180.51 - 184.22) | 196.47 (194.97 - 197.96) |

**Supplementary Table 5:** Age adjusted mortality rates per 100,000 deaths stratified by urbanization in adults aged 35+ in the United States, 1999 to 2020

| **Table 6:** State wise age adjusted mortality rates per 100,000 deaths in adults aged 35+ in the United States, 1999 to 2020 | | |
| --- | --- | --- |
| **State** | **AAMR per 100,000 deaths (95% CI)** | **Percentage of Deaths** |
| Alabama | 138.96 (138 - 139.92) | 1.07% |
| Alaska | 128.83 (125.45 - 132.2) | 0.08% |
| Arizona | 171.63 (170.71 - 172.55) | 1.76% |
| Arkansas | 180.07 (178.71 - 181.44) | 0.88% |
| California | 209.85 (209.41 - 210.3) | 11.17% |
| Colorado | 151.64 (150.56 - 152.71) | 1.01% |
| Connecticut | 176.46 (175.29 - 177.63) | 1.17% |
| Delaware | 216.6 (213.89 - 219.31) | 0.33% |
| District of Columbia | 133.97 (131.19 - 136.75) | 0.12% |
| Florida | 201.28 (200.77 - 201.79) | 7.93% |
| Georgia | 150.68 (149.89 - 151.48) | 1.85% |
| Hawaii | 131.16 (129.5 - 132.83) | 0.32% |
| Idaho | 164.32 (162.42 - 166.23) | 0.38% |
| Illinois | 197.4 (196.7 - 198.11) | 3.99% |
| Indiana | 222.93 (221.88 - 223.98) | 2.29% |
| Iowa | 221.42 (220.03 - 222.82) | 1.30% |
| Kansas | 197.65 (196.2 - 199.1) | 0.95% |
| Kentucky | 200.6 (199.38 - 201.83) | 1.36% |
| Louisiana | 159.92 (158.83 - 161.02) | 1.09% |
| Maine | 189.81 (187.87 - 191.75) | 0.49% |
| Maryland | 177.09 (176.07 - 178.11) | 1.54% |
| Massachusetts | 162.41 (161.57 - 163.25) | 1.91% |
| Michigan | 227.65 (226.82 - 228.49) | 3.80% |
| Minnesota | 168.93 (167.94 - 169.93) | 1.48% |
| Mississippi | 182 (180.56 - 183.43) | 0.82% |
| Missouri | 216.17 (215.13 - 217.21) | 2.20% |
| Montana | 143.7 (141.66 - 145.73) | 0.25% |
| Nebraska | 195.47 (193.69 - 197.24) | 0.62% |
| Nevada | 138.49 (137.06 - 139.91) | 0.50% |
| New Hampshire | 192.15 (190.05 - 194.25) | 0.43% |
| New Jersey | 222.25 (221.39 - 223.12) | 3.34% |
| New Mexico | 155.88 (154.29 - 157.47) | 0.49% |
| New York | 284.87 (284.21 - 285.52) | 9.49% |
| North Carolina | 180.05 (179.26 - 180.84) | 2.61% |
| North Dakota | 208.38 (205.48 - 211.28) | 0.27% |
| Ohio | 270.22 (269.4 - 271.05) | 5.38% |
| Oklahoma | 235.43 (234.01 - 236.84) | 1.41% |
| Oregon | 185.25 (184.05 - 186.45) | 1.22% |
| Pennsylvania | 214.88 (214.22 - 215.55) | 5.34% |
| Rhode Island | 236.58 (234.14 - 239.03) | 0.48% |
| South Carolina | 176.57 (175.45 - 177.68) | 1.29% |
| South Dakota | 191 (188.45 - 193.55) | 0.29% |
| Tennessee | 233.4 (232.31 - 234.49) | 2.32% |
| Texas | 187.24 (186.69 - 187.79) | 5.84% |
| Utah | 142.1 (140.55 - 143.66) | 0.42% |
| Vermont | 186.98 (184.06 - 189.9) | 0.21% |
| Virginia | 149.42 (148.61 - 150.22) | 1.75% |
| Washington | 176.7 (175.76 - 177.64) | 1.81% |
| West Virginia | 291.14 (289.07 - 293.21) | 1.00% |
| Wisconsin | 184.2 (183.22 - 185.18) | 1.81% |
| Wyoming | 167.75 (164.55 - 170.94) | 0.14% |

**Supplementary Table 6:** State wise age adjusted mortality rates per 100,000 deaths in adults aged 35+ in the United States, 1999 to 2020
